# Fitting HIV Prevalence 1981 Onwards for Three Indian States Using the Goals Model and the Estimation and Projection Package

**DOI:** 10.1371/journal.pone.0164001

**Published:** 2016-10-06

**Authors:** Tarun Bhatnagar, Tapati Dutta, John Stover, Sheela Godbole, Damodar Sahu, Kangusamy Boopathi, Shilpa Bembalkar, Kh. Jitenkumar Singh, Rajat Goyal, Arvind Pandey, Sanjay M. Mehendale

**Affiliations:** 1 National Institute of Epidemiology, Indian Council of Medical Research, Chennai, Tamil Nadu, India; 2 International AIDS Vaccine Initiative, New Delhi, India; 3 Avenir Health, Glastonbury, Connecticut, United States of America; 4 Department of Epidemiology, National AIDS Research Institute, Indian Council of Medical Research, Pune, Maharashtra, India; 5 National Institute of Medical Statistics, Indian Council of Medical Research, New Delhi, India; Hokkaido University Graduate School of Medicine, JAPAN

## Abstract

Models are designed to provide evidence for strategic program planning by examining the impact of different interventions on projected HIV incidence. We employed the Goals Model to fit the HIV epidemic curves in Andhra Pradesh, Maharashtra and Tamil Nadu states of India where HIV epidemic is considered to have matured and in a declining phase. Input data in the Goals Model consisted of demographic, epidemiological, transmission-related and risk group wise behavioral parameters. The HIV prevalence curves generated in the Goals Model for each risk group in the three states were compared with the epidemic curves generated by the Estimation and Projection Package (EPP) that the national program is routinely using. In all the three states, the HIV prevalence trends for high-risk populations simulated by the Goals Model matched well with those derived using state-level HIV surveillance data in the EPP. However, trends for the low- and medium-risk populations differed between the two models. This highlights the need to generate more representative and robust data in these sub-populations and consider some structural changes in the modeling equation and parameters in the Goals Model to effectively use it to assess the impact of future strategies of HIV control in various sub-populations in India at the sub-national level.

## Introduction

Mathematical modeling and computer simulation are powerful tools to evaluate impact of intervention programs, formulate and inform health policy decisions and decide the direction for advocacy efforts. Various simulation models have been used to understand the dynamics of the human immunodeficiency virus (HIV) epidemic, and investigate the impact of biomedical and behavioral interventions on its progression [1–15]. A few models have been designed and used to specifically capture the dynamics of India’s HIV epidemic [16–21]. Estimations Projection Package (EPP) is recommended by the Joint United Nations Program on HIV and AIDS (UNAIDS) globally to estimate and project national HIV epidemics [22, 23]. The Goals Model, part of the Spectrum System of Policy Models, (www.avenirhealth.org) has been used extensively for estimating changes in HIV prevalence/ incidence, evaluating the coverage of care and treatment services, allocating resources between prevention and care programs, setting priorities for high-risk populations and assessing the potential impact of HIV vaccine [24–32].

The HIV epidemics in the Indian states of Andhra Pradesh, Maharashtra and Tamil Nadu are considered to have ‘matured’ and have also shown a significant decline in HIV prevalence among high-risk populations and also in the general population, albeit at different levels [33, 34, 35]. Prevention policies and programs implemented in these states include awareness programs, targeted intervention among high risk groups, condom promotion, access to voluntary testing as well as treatment of sexually transmitted infections (STI) and HIV care and support. These have been scaled up over time, with a documented positive impact [36, 37].

The Goals Model provides a tool to test the potential additive impact of interventions on reducing new HIV infections. We hypothesized that the newer HIV prevention methods (vaccine, microbicides, pre-exposure prophylactic drugs, circumcision etc.) are likely to further reduce HIV disease burden in the states of Andhra Pradesh, Maharashtra and Tamil Nadu in India. We employed the Goals Modal to generate HIV prevalence curves for the states of Andhra Pradesh, Maharashtra and Tamil Nadu in India, compared those with HIV prevalence curves generated by the EPP software and have discussed methodological concerns and utility of the Goals Model in the context of available data sources at state level.

## Methods

### Structure of the Goals Model

This Model is built on the basis of specific determinants of transmission of HIV and a risk-group structure. It simulates the adult, gender dis-aggregated, sexually active population (15–49 years) in various risk groups. Married and/or monogamous people are categorized as low-risk heterosexuals and married people with multiple casual partners as medium-risk heterosexuals. People with multiple partners (female sex workers (FSW) and their clients), injecting drug users (IDU) and men who have sex with men (MSM) are categorized as high-risk groups. Every person entering the Model population is assumed to be HIV-negative and to remain uninfected when not sexually active. Any person who may adopt low-risk behavior is appropriately tracked in the Model. The Goals Model decides the probability of acquiring new HIV infection by characteristics of the index person and the partner, along with the transmission dynamics of the partnership.

### Input parameters

Input parameters in the Goals model included demographic (population by age and sex, total fertility rate, sex ratio at birth, life expectancy at birth—by gender, net migration), epidemiological (HIV prevalence by risk groups, STI prevalence by gender), and transmission-related and risk group-wise behavioral parameters (distribution of the population by risk group, proportion of sex acts protected by condom use, number of partners per year, number of sexual contacts per partner per year, age at first sex by gender and % married by gender). The parameter values for respective years and their sources are given in Table 1.

**Table 1:** Values and sources for input parameters in the Goals Model used for modeling in Tamil Nadu, Maharashtra and Andhra Pradesh.

Parameters	Tamil Nadu		Maharashtra		Andhra Pradesh	
	Year	Value	Year	Value	Year	Value
**Epidemiology**						
*Transmission per act*						
Transmission of HIV per act (female to male)	NA	0.0007[38]	NA	0.0007[38]	NA	0.0007[38]
Transmission multiplier for male to female	NA	1[38]	NA	1[38]	NA	1[38]
Transmission multiplier for STI	NA	5[38, 39]	NA	5[38, 39]	NA	3[38, 39]
Transmission multiplier for MSM contacts	NA	3(Default)	NA	4 (Default)	NA	3 (Default)
*Relative infectiousness by stage*						
Primary Infection	NA	9.17[40]	NA	7[40]	NA	9.17[40]
Months in Primary Stage	NA	3[40]	NA	3[40]	NA	3[40]
Asymptomatic stage	NA	1	NA	1	NA	1
Symptomatic stage (no ART)	NA	8[41]	NA	4[41]	NA	7.27[40]
Symptomatic stage (with ART)	NA	0.04[41]	NA	0.06[41]	NA	0.04[41]
*Transmission reduction (0–100%)*						
Condom efficacy	NA	80[42]	NA	80[42]		80[42]
Reduction in male susceptibility when circumcised	NA	60[43]	NA	60[44,45]	NA	60[34,35]
*Initial pulse*						
Size of initial pulse of infection (0–0.01)	NA	0.001 (Assumption)	NA	0.003 (Assumption)	NA	0.001 (Assumption)
**Blood Transfusion**						
New infections	1981–2017	0 (Assumption)	1981–2017	0 (Assumption)	1981–2017	0 (Assumption)
**Behavior**						
*% of population in each group*						
*Males*						
Not sexually active	2009	24.8[46]	2005	35.9[47]	2005	25.73[48]
Low risk heterosexual	1981–2017	57.4 (Calculated)	1981–2017	47.68 (Calculated)	1981–2017	61.75 (Calculated)
Medium risk heterosexual	2009	10.45[46]	2009	7.74[46]	2005	6[46]
High risk heterosexual	2009	4.93[46]	2009	4.93[46]	2009	6.5[46]
Injecting drug user	2009	0.02[46]	2009	0.01[46]	2009	0[46]
Men who have sex with men	2009	2.4[46]	2009	3.74[46]	2009	0.02[46]
*Females*						
Not sexually active	2009	23.3[46]	2005	21.9[47]	2005	16.33[48]
Low risk heterosexual	2009	65.89 (Calculated)	2009	70.06 (Calculated)	2009	76.97 (Calculated)
Medium risk heterosexual	2009	10.45[46]	2009	7.74[46]	2009	4.5[46]
High risk heterosexual	2009	0.36[46]	2009	0.3[46]	2009	2.2[46]
Injecting drug user	2009	0[46]	2009	0[46]	2009	0[46]
*Injecting drug user*						
Force of infection: Males (0–1)		NA		NA	1981, 2010	0.01, 0.2 (Assumption)
Force of infection: Females (0–1)		NA		NA	1981	0.19 (Assumption)
% of IDU sharing needles (0–100%)		0	2006, 2007, 2009, 2010	35[49], 6[50], 41[51],14[50]	2009	40[48]
**Behavior**						
*Condom Use*						
Low risk heterosexual	2002, 2005	2[52], 3.2[53]	1981	0 (Assumption)	1981	0 (Assumption)
Medium risk heterosexual	2002, 2005	34.2[52], 41.2[53]	1981	0 (Assumption)	1981	0 (Assumption)
High risk heterosexual	2007, 2009	75.4[49], 91.5[51]	2001, 2006	87.7, 98.3[50]	2009	32[51]
Men who have sex with men	2007, 2009	71.1[49], 85.6[51]	2009	70–80[51]	1981	0 (Assumption)
*No*. *of partners*						
*Males*						
Low risk heterosexual	1981–2017	1 (Default)	1981–2017	1 (Default)	1981–2017	1 (Default)
Medium risk heterosexual	1981–2017	5 to 1 (Default)	1981–2017	5 to 1 (Default)	1981–2017	5 (Default)
High risk heterosexual	2007, 2009	10[49],16[51]	2006, 2009	24[49],12[51]	1981–2017	8 (Default)
Men who have sex with men	2009	34[51]	1981–2017	12 to 3 (Default)	1981–2017	8 (Default)
*Females*						
Low risk heterosexual	1981–2017	1 (Default)	1981–2017	1 (Default)	1981–2017	1 (Default)
Medium risk heterosexual	1981–2017	5 to 1 (Default)	1981–2017	5 to 1 (Default)	1981–2017	5 to 1 (Default)
High risk heterosexual	2007, 2009	187[49], 62[51]	2006, 2009	468[49], 520[51]	1981–2017	175 (Default)
*Sex Acts*						
Low risk heterosexual	1981–2017	1 (Default)	1981–2017	1 (Default)	2009	35[46]
Medium risk heterosexual	1981–2017	5 to 1(Default)	1981–2017	5 to 1 (Default)	2009	25[47]
High risk heterosexual	2007, 2009	15[49], 3.7[51]	1981–2017	9 (Default)	2009	11[47]
Men who have sex with men	2009	7[51]	1981–2017	8 (Default)	2009	8[47]
*Age at first sex*						
Males	2002, 2005	20 years[52, 53]	1998–2000, 2005	19[54], 24.4 years[47]	2005	21[48]
Females	2002, 2005	19 years[39,40]	1993–2002, 2005	16[55], 7.8 years[47]	2005	16.3[48]
*% married*						
*Males*						
Low risk heterosexual	1981–2017	100 (Default)	1981–2017	100 (Default)	1981–2017	100 (Default)
Medium risk heterosexual	2006	59.2[46]	2009	92[46]	1981–2017	75 (Default)
High risk heterosexual	2009	64[51]	2009	70.5[46]	2009	60[51]
Injecting drug user	1981–2017	66 (Default)	2010	71[51]	2009	56[51]
Men who have sex with men	2009	24.1[51]	2010	71[51]	2009	45.6[51]
*Females*						
Low risk heterosexual	1981–2017	100 (Default)	1981–2017	100 (Default)	1981–2017	100 (Default)
Medium risk heterosexual	2006	73.6[56]	2009	92[46]	1981–2017	85 (Default)
High risk heterosexual	2009	95.6[51]	2010	53[51]	1981–2017	50 (Default)

### Modeling process and output

We configured the Goals Model for the period 1981–2017, as the first case of Acquired Immuno-deficiency Syndrome (AIDS) was reported from India in 1986 [57]. After entering the data available for this period we used the duplicate or linear interpolation functions of the Model applying logic or evidence for the intermediate time points. The Goals Model is linked to a demographic projection model (DemProj) and the AIDS Impact Model (AIM) within Spectrum. DemProj module was used to perform demographic projection by age and sex, based on the assumptions of past, current and future fertility, mortality and migration. The Estimation and Projection Package (EPP) within the AIM module was used to estimate HIV prevalence and generate curves for each of the risk groups using programmatic and epidemiological data available from an estimation exercise done earlier for each state by the National/Regional Working Group on HIV Estimations [37, 58]. For model fitting we compared the HIV prevalence estimated by the Goals Model with the epidemic curve generated for all adults and by risk groups by the EPP.

## Results

**Fig 1:**
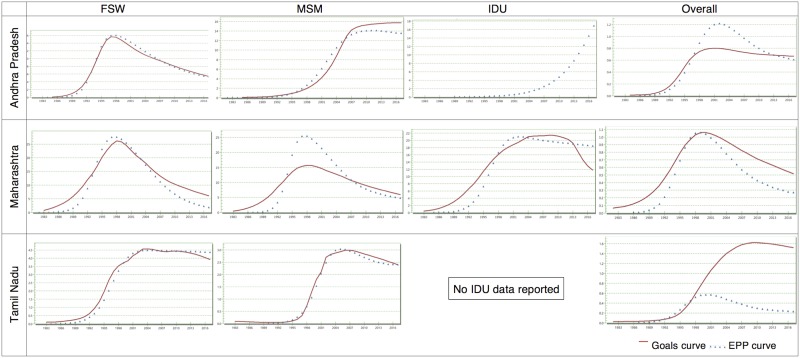
Adult HIV prevalence curves for Andhra Pradesh, Maharashtra and Tamil Nadu generated by the Goals Model and EPP/AIM.

Fig 1 shows the HIV prevalence curves, for FSW, MSM, IDU and all adults, generated by the Goals Model and the EPP for the three states.

For Andhra Pradesh, the Goals Model curve for FSW followed the pattern of the EPP curve. The Goals Model curve for MSM had a reasonably good fit with the EPP curve with prevalence being slightly lower between 1998 and 2005 and higher 2008 onwards. The Goals Model did not generate a curve for IDU, probably due to insufficient data. Overall, the Goals Model curve could fit the initial rise until 1997 and then had a much lower peak. It plateaued earlier than the EPP curve.

For Maharashtra, the FSW prevalence curve generated by the Goals Model rose earlier followed by a lower decline (2005 onwards), the MSM curve showed higher prevalence between 1983 and 1993 followed by a much lower peak and the IDU curve showed much higher prevalence prior to 1997 and between 2004 and 2013 followed by a drastic decline in comparison to the EPP curve. For the total prevalence, compared to the EPP curve, the Goals curve showed a higher prevalence as it rose from 1983 to 1996 and then declined 2000 onwards.

In case of Tamil Nadu, the Goals Model curve had a reasonably good fit with the EPP curve for both FSW and MSM, except for the steeper decline for FSW (after 2011) and a flatter curve for MSM (2008 onwards). As per the EPP curve the total adult HIV prevalence started rising around 1990–1991, gradually peaked in 2000 with a prevalence of around 0.6% followed by a gradual decline to 0.3% in 2010 and flattening around 0.2% thereafter. The total adult prevalence curve generated by the Goals Model showed a steep rise beginning 1993–1994 until 2008–2009 (1.6%) with a marginal decline and plateauing thereafter. Overall, the Goals Model curve for all adults in Tamil Nadu did not fit the EPP curve.

## Discussion

Our analysis has shown that for high-risk populations, the HIV prevalence trends simulated by the Goals Model match reasonably well with the trends derived from surveillance data with the EPP model. However, trends for the remaining populations differed between the two approaches.

While comparing the outputs of the Goals and EPP models it is worthwhile considering the differences in the structure, input parameters and computing approaches of the two models within the Spectrum software. The EPP is a tool for country-level estimation and short-term projection of HIV/AIDS epidemics based on observed HIV surveillance data on prevalence. The EPP application does not distinguish between urban and rural zones and does not accommodate sub-national epidemics resulting from regional variations. EPP inputs include data on mother to child transmission, adult and child anti-retroviral treatment (ART) coverage, fixed values of survival probability and effect of ART and total fertility rate. In comparison, the Goals Model allows local adaptations in the model as per the local epidemic and program data. Whenever local values are not available, inbuilt default values are used [59]. The Goals Model inputs also include data regarding sexual behavior by risk group, demographic data, base year human capacity, and assumptions about types of care and prevention activities/ program data, in four different epidemic scenarios. Model building in the Goals also considers behavioral and epidemiological factors, which is not the case in the EPP.

The curve fitting approach of the EPP uses antenatal clinic surveillance data and generally showed significant declines in prevalence since 2000. The simulations with the Goals Model, however, showed much less decline. In the Goals Model this population is divided into low risk (single partner) and medium risk (multiple partners), with the majority of the population in the low risk category. We hypothesize that behaviors such as coital frequency and condom use in the low risk population have changed little since 2000. As a result the Goals Model estimated that prevalence declined very slowly. Although the prevalence in the medium risk population is likely to decline more rapidly as a result of increased condom use and reduced prevalence of STIs, and, perhaps, declines in the number of partners, the size of this population is too small to affect the overall trend much. The two approaches would result in a comparable prevalence if the medium risk population represented a much larger proportion of the total population.

Despite a good fit for the high-risk populations, the prevalence pattern among low and medium risk populations was different compared to the Goals Model projection. If we disregard the low/medium risk group projections from EPP, then we will have to consider fitting to a different pattern for overall prevalence. To match the overall prevalence pattern we would be required to either increase the proportion of the population in the medium risk group so that those dynamics would have more influence on the overall trend, or change the dynamics of the low risk population. This would depend on the change in the factors over time that could have affected the low risk population, such as number of acts per partner, condom use and STI prevalence. Consequently, the prevalence in the low risk population reflects a lagged relationship to the prevalence in the higher risk populations. Assuming that there has been a very little change in factors affecting the low risk population, it would require an increase in the proportion of the population classified as medium risk to fit the curve in the model. Conversely, we may assume that the prevalence pattern from the EPP for low risk populations is not correct and accept the curve generated by the Goals Model. The major bottleneck to justify either of the scenarios is the limited availability of behavioral data for medium and low risk population groups in all the three states, where the focus of research as well as interventions has been on high risk population groups because of the concentrated nature of the HIV/AIDS epidemic. Current estimates are based on self-reported behaviors. Studies show that risky behaviors are often under-reported in surveys [60]. So it may be possible that the size of the medium risk population is actually much larger. It is difficult to confirm or reject the hypothesis that many more people have multiple partners than reported in surveys for the want of adequate behavioral data.

Adjustments can be made in several parameters of the Goals Model in an attempt to generate HIV prevalence curve comparable to the EPP-based curve. The adjustments include 1) modifying the ‘size of initial pulse of infection’ to change the initial slope of the epidemic curve, 2) changing the ‘epidemic start year’ to earlier or later year, 3) changing ‘transmission possibility of HIV per act (female to male)’ and ‘primary infection stage’ to change the height of the prevalence curve, 4) altering ‘STI prevalence’ and the trend in ‘condom use’, 5) changing ‘IDU force of infection (male and female)’ to fit the IDU prevalence trend, 6) adjusting ‘number of partners’ and ‘acts per partner’ by risk groups, and 7) changing the ‘% married’[59].

The overall Goals prevalence curve in Maharashtra did not decline as much after 2005 as did the EPP curve. Increase in the number of HIV positive people in the low risk category was offsetting the overall decline in the other risk groups. Additionally, the model had sharply declining prevalence among the medium risk population but among the low risk population it was rising and reaching a plateau. For the prevalence to be lower in the low risk population, the proportion married should be low. However, this change in model parameter will lower the proportion of the low risk population having sex with high-risk populations and thus reduce the transmission of infection from higher to lower risk populations. Similarly, reducing the frequency of sex or increasing condom use for the low risk population will reduce the likelihood of exposure. The last two options are justifiable for the low risk people only in case of known HIV positive partners and cannot be universally applied. Limitations of any model become more and more apparent as the complexity of transmission increases. It is difficult to replicate all the complexities of HIV transmission and lesser characterized factors impacting the real life epidemic in a model, such as duration of each unprotected sex act, volume of the semen ejaculated in the vagina and immediate post-intercourse washing up practices, for which data is usually not available.

There are several issues regarding the structure and behavior of the Goals Model that warrant attention in the Indian context. There is only limited flow of people between the risk groups as formulated in the Goals Model. The ‘not sexually active’ population could also show some HIV prevalence. For example, at the age of 15 years the HIV positive children are considered as adults and are allocated to the various risk groups. So if 30% of all adults are 'not sexually active' then 30% of HIV+ children at age 15 years will be allocated to the 'not sexually active' population group [59]. In reviewing this dynamics we discovered an error in the code that has been corrected in the newer version of the model. However, major changes in the modeling outputs are not expected because of such a small correction. Secondly, in real life an individual may be in different risk groups at different times. These changes need to be expressed through the average number of partners for each risk group. The Goals Model is limited in its ability to capture this dynamism. Thirdly, risk is a continuous variable but as per the Model specification we had to categorize it into six groups for males and five for females. Ideally, it would be more realistic to consider many more groups that would better reflect the risk continuum. However, there is no data to support more risk categories in the Model. This implies that the values needed in the model to get a good fit would vary from those reported in surveys or available EPP data. Lastly, the task any modeler would face would be deciding whether the resulting model—if it fits the historical epidemic dynamics well—would be useful to simulate projections for the future impact of interventions and prevention methods. In general it seems more acceptable for biomedical interventions, such as ART, pre-exposure prophylaxis and vaccines, which act on the probability of transmission, than for behavioral interventions, such as outreach, care and treatment, which influence risk through behavior change.

Some of the transmission-related parameters used for fitting HIV prevalence curves were different for the three states. It is generally considered that biological factors and vulnerability need not vary within the country. We were unable to reconcile to one set of values that worked for all the states in terms of fitting the model. The values of epidemiological parameters used for fitting HIV prevalence curve were also different across the three states. This points to the possibility that biological factors may be different in each state in addition to the likely variability in risk behaviors for HIV acquisition. Further, the probability of HIV transmission per sex act depends on some factors not included separately in the model, such as the proportion of acts that involve anal sex (for heterosexual contacts), use of lubricants or desiccants, roughness of sex, etc., although their impact at population level warrants investigation. Similarly, the STI multiplier depends on the types and prevalence of STIs. The model also discounts on parent to child transmission and assumes that all who are not sexually active i.e., children below 15 years of age are sero-negative. The MSM multiplier depends on the proportion of anal and oral sex acts. The primary stage multiplier depends on the degree of partner concurrency. Addition of these behavioral parameters influencing HIV transmission in the mathematical equation for estimating HIV prevalence and incidence in the Goals Model may be considered in order to improve the modeling results and take into account behavioral differences across regions. For more accurate modeling results, the extent of inter-state variations with respect to such factors will have to be further explored.

Changing some of the biological parameters, such as probability of transmission per act or primary stage multiplier, affected all population groups except IDU. Changing only behavioral parameters for MSM or FSW had some effect on low-risk populations because some of them had high-risk partners. Other changes, such as the number of sex acts for MSM did not have any effect on FSW prevalence. Thus, in Tamil Nadu model we first tried to fit the HIV prevalence curve for FSWs alone and then for MSM. After fitting the curve for MSM we found that the curve for FSW changed.

In Maharashtra we got a reasonably fit for high-risk groups, primarily by adjusting the values of number of partners, albeit without any evidence-based justification. In Tamil Nadu and Maharashtra initially we tried to fit the prevalence curve for FSW and MSM using behavioral data available from different sources for different time periods and interpolating the rest, but could not get a suitable fit. We then arbitrarily changed the values of these parameters such that we got a near fit. In Andhra Pradesh we could achieve a reasonable fit keeping the values for number of partners and number of sex acts per partner for FSW as well as MSM constant for all the years. However, these parameter values had to be kept different across the years to obtain a good fit in Tamil Nadu and Maharashtra, which appears rather unreal. Also, different combinations of parameter values yielded very similar curves. Changing the probability of transmission per act had a similar effect to changing the primary stage multiplier. Changing STI patterns or the condom patterns in some cases also showed similar effects. As each indicator is a factor in the transmission equation, changes in one factor can offset others.

The published data from various surveys in the three states that were used as input values in the Goals Model also has its limitations. The questions asked in surveys may not always correspond exactly to the indicators used in the model. The participants may not always respond correctly, either intentionally or unintentionally, which could affect the veracity of the data. For this exercise the data for epidemiological and behavioral parameters is available 2005 onwards, while the base year for the analysis is 1981. There is lack of data in the early part of the epidemic during the eighties and nineties. Hence, we had to fill the missing data points by changing the values of some of the key parameters—number of partners, condom use, and proportion married. However, these data adjustments were not in line with the current understanding of programmatic and research evidence and available data on HIV epidemiology and behavior in the three states. Availability of relevant input parameter data for corresponding time points as per the model requirement was a major challenge in achieving the objectives of this analysis. Further, the available data used for parameter values for making the projections have been generated from program interventions and research studies focused in limited geographical areas within the States and may not fully reflect the epidemic status in the State. We are hence left unsure whether simulation models, such as the Goals Model, can be used to assess the impact of future strategies in India.

HIV transmission models have been continuously evolving to analyze trends of epidemics in countries and regions and assess potential impact of emerging interventions. Their contextualization and validation in various geographic regions with HIV epidemics of different nature is an important process. In order to employ the Goals Model to evaluate the impact of HIV interventions in the Indian context, there is an imperative need for more research studies representative at the state level to generate data on transmission parameters and behavioral patterns. Such a modeling exercise in the future would benefit from more dependable and robust program data coupled with routine analysis for effective and timely actions. It would be worthwhile to consider structural changes in the modeling equation and parameters in the Goals Model, as discussed above, to make it more responsive and adaptable to differing HIV epidemic scenarios and behavioral patterns, especially while modeling at the sub-national level.
